# Effects of individual immigrant attitudes and host culture attitudes on doctor-immigrant patient relationships and communication in Canada

**DOI:** 10.1186/s12939-015-0250-3

**Published:** 2015-10-29

**Authors:** Amanda Whittal, Ellen Rosenberg

**Affiliations:** Bremen International Graduate School of Social Sciences, Jacobs University, Campus Ring 1, South Hall, 28759 Bremen, Germany; McGill University, St. Mary’s Hospital, 3830 Lacombe Avenue, Montreal, QC H3T 1M5 Canada

**Keywords:** Acculturation orientation and health, Cultural communication, Health communication, Doctor-patient communicaiton, Culture and health

## Abstract

**Introduction:**

In many countries doctors are seeing an increasing amount of immigrant patients. The communication and relationship between such groups often needs to be improved, with the crucial factor potentially being the basic attitudes (acculturation orientations) of the doctors and patients. This study therefore explores how acculturation orientations of Canadian doctors and immigrant patients impact the doctor-patient relationship.

**Methods:**

*N* = 10 participants (five doctors, five patients) participated in acculturation orientation surveys, video recordings of a regular clinic visit, and semi structured interviews with each person. Acculturation orientations were calculated using the Euclidean distance method, video recordings were analyzed according to the Verona Coding System, and thematic analysis was used to analyze the interviews. Interviews were used to explain and interpret the behaviours observed in the video recordings.

**Results:**

The combined acculturation orientations of each the doctor and immigrant patient played a role in the doctor-patient relationship, although different combinations than expected produced working relationships. Video recordings and interviews revealed that these particular immigrant patients were open to adapting to their new society, and that the doctors were generally accepting of the immigrants’ previous culture. This produced a common level of understanding from which the relationship could work effectively.

**Conclusion:**

A good relationship and level of communication between doctors and immigrant patients may have its foundation in acculturation orientations, which may affect the quality of care, health behaviours and quality of life of the immigrant. The implications of these findings are more significant when considering effective interventions to improve the quality of doctor-patient relationships, which should have a solid foundational framework. Our research suggests that interventions based on understanding the influence of acculturation orientations could help create a basic level of understanding, and therefore improved interaction between doctors and immigrant patients.

## Introduction

In response to the current societal shifts moving us into an increasingly globalized world, a deeper understanding of diverse individuals and their behaviours is required. A key area where such an understanding is lacking is in the relationship and communication between doctors and immigrant patients, which is often challenging and can influence the patient’s health [1].

Canada has long been established as a multicultural country, and immigration continues to increase. In 2006, Canada’s immigrant population made up 1/5 of the country’s population, and is expected to reach at least 1/4 of the population by 2031 [2]. Canada currently also has a low national birthrate and an increasing reliance on immigrants, who are responsible for 2/3 of Canada’s 5.4 % growth [3].

The growing rates of immigration, and the increasing reliance on immigrants for a strong workforce and economy highlight the importance of existing research, which has found that most immigrants, though a valuable part of the society, are in many aspects not treated as equal to the native population, such as in the workforce [4] and in health care [5].

Inequality in immigrant health care is a particular area of concern, as poorer quality of care for immigrants is a common occurrence [5]. Numerous researchers point out, however, that it is not well understood *why* such disparities exist [6–8].

As a result of growing immigration, there is not only an increased number of people requiring use of the health care system, but it also becomes more difficult to meet peoples’ needs when their values and background are increasingly diverse and not well understood [9]. This can been seen in findings of a direct association between social integration (i.e. an immigrant’s connections within a new society in terms of marital status, voluntary membership in associations, and interactions with friends/relatives) and health: better social integration is associated with lower blood pressure and fewer depressive symptoms, and vice versa [10, 11]. Although it is understood that culture (i.e., the learned behaviours, beliefs, and attitudes characteristic of a particular population) plays a crucial role in immigrant health, there is a lack of theoretical models that can accurately connect culture with individual (i.e. doctor-patient interactions) and biological (i.e. health outcomes) elements [9, 11]. Still, a substantial amount of evidence exists to support the fact that culture can influence the quality of healthcare received by immigrant patients via a complex and poorly understood process [12].

As an example, studies utilizing recorded clinic visits or interviews found that physicians native to a country tend to show less empathy toward immigrant patients, and gaps of misunderstanding exist in communication [7, 13–15]. This may not necessarily be due to conscious discrimination, but rather the setting in which the interaction occurs: doctors are under extreme time pressure, high cognitive load, and stress. This may make them more likely to draw on stereotype assumptions when they come into contact with immigrant groups, rather than assessing individual characteristics, which requires much more time and energy [16]. Unfortunately, such disparities in understanding and communication have been linked to poorer quality of care provided [5].

Previous studies have found that communication between doctors and immigrant patients is less effective than with native patients, containing more misunderstandings and less patient compliance [17, 18]. The reasons for such misunderstandings, however, are not clearly understood. It is therefore essential to investigate this, to understand both how an individual with a different culture background is affected by different perspectives, *and* how the attitude of the healthcare providers can have an effect.

This study therefore explores how individual cultural attitudes influence the relationship between native doctors and immigrant patients, which may in turn influence the quality of care provided, and the resulting health behaviours and quality of life of the patient.

Acculturation orientation (AO) has been chosen as the measurement of attitudes, because although it would be ideal for doctors to have a basic knowledge of the different backgrounds of each immigrant patient they treat, this is a high and unrealistic demand. Physicians already experience high cognitive load and stress [19]. It is therefore more practical to measure individual cultural attitudes at a more fundamental level. Assessing AO examines the *expectations* of the physician and immigrant, rather than the details of cultural values and beliefs. Because doctors’ expectations and perceptions of patients have been shown to influence the doctor-patient relationship [20], this may provide a more basic way of creating a solid foundation for a working interaction.

### Acculturation orientation

AO is based on Berry’s acculturation model [21]. This model provides a well rounded way of operationalizing one’s cultural ‘attitude’, as it classifies an individual’s AO when moving to a new ‘host’ culture into four categories, seen in Table 1 below.^1^

**Table 1:** Berry’s acculturation model

		Cultural maintenance (Of Immigrant OR Host Culture)	
		High	Low
Contact and Participation (Of Immigrant OR Host Culture)	High	Integration	Assimilation
		Interest in maintaining one’s original culture while also participating in daily and social activities of the dominant group and with other ethnic and cultural groups	Individual does not wish to maintain his/her cultural identity and seeks daily interactions with other cultures
	Low	Separation	Marginalization
		Individuals place a high value on holding onto their original culture and avoid interaction with others	Little possibility or interest in having relationships with others and little interest in or possibility of cultural maintenance (due primarily to experiences with discrimination or instituationalized, forced separation from others).

(Berry, [21])

Based on the above model, the Interactive Acculturation Model (IAM) was created to include *host culture* acculturation orientations, as part of a dynamic interplay between host society members and immigrants [22]. The model seeks to predict the types of relationships that would be formed between host culture members and immigrants, based on their respective AOs. It does so by taking the orientation of the host country, and that of the immigrant, and predicting what kind of relationship will be formed.

The IAM was intended for a societal level scale in terms of host culture *group* and immigrant *group.* It was further modified by Kazarian&Evans [23] to apply specifically to healthcare settings, a form in which it is intended to be interpreted on the individual level.

This model is conceptualized in Table 2. It refers specifically to the relational outcomes of health consumer and health professional orientations, and which combinations are predicted to produce favourable and unfavourable doctor-patient relationships.

**Table 2:** Modification of Berry’s model: the health consumer/health practitioner model

	Health consumer			
Health professional	*Integration*	*Assimilation*	*Separation*	*Individualism (Marginalization)*
*Integration*	Consensual	Problematic	Conflictual	Problematic
*Assimilation*	Problematic	Consensual	Conflictual	Problematic
*Separation*	Conflictual	Conflictual	Conflictual	Conflictual
*Individualism*	Conflictual	Conflictual	Conflictual	Conflictual

(Kazarian & Evans [23])

The AO of the health professional refers to his or her belief about whether the immigrant patient should be assimilated, integrated, separated or marginalized into the new culture.

Consensual, problematic, and conflictual refer to the type of healthcare provider-patient relationship that can be expected from various combinations of AOs of native doctors, and immigrant patients. Consensual relationships suggest a shared understanding. Problematic relationships suggest no direct conflict in opinions, but also no common understanding. Conflictual relationships suggest a direct conflict of opinions.

This is the only model to our knowledge that predicts how AOs relate to the relationship of health consumers and health professionals. Since implementation of research findings in the medical world is often haphazard, a model framework is useful [24]. Since this model has a strong theoretical basis [23], but has not yet been tested, this study uses it to observe if its proposed combinations of AOs do in fact interrelate with the doctor-patient relationship, as predicted, in a real-world setting.

The health consumer/health professional model guided the development of the following research questions:

1. Does the AO of doctors and immigrant patients influence their relationship, based on the health consumer/health practitioner model?
2. How do the AOs of physicians native to a country towards immigrant patients, and the AOs of these immigrant patients, interrelate with the quality of the doctor-patient relationship/experiences of both individuals?

## Methods

### Recruitment

The study received ethical approval from the ethics board at St. Mary's Hospital, in the culturally diverse city of of Montreal, Canada. The researcher was granted access to the hospital to invite family doctors face-to-face to participate in the study. Those who agreed provided a list of their immigrant patients, who were invited to participate via phone call.

### Population

*N* = 10: 5 doctors (4 females, 1 male), each with one immigrant patient (4 females, 1 male) took part in a survey, videotaped clinic visits, and semi-structured interviews. The patients had a migration background (immigrated to Canada after the age of 16) and the doctors did not (trained and practicing in Canada). In total, five clinic visits were videotaped, and 10 interviews were conducted (5 doctors, 5 patients). Following the 10 interviews, no new information was being obtained, so the recruitment process was stopped at 5 cases due to saturation.

### Procedure

All video recordings and interviews occurred in Saint Mary’s Hospital. Details of the procedure were explained to doctors and patients, and each signed a consent form prior to commencement of the study.

Patients and doctors each filled in a two page questionnaire to determine their AOs. For patients, this referred to their orientations towards the host culture (Canada), with questions including such items as “it is important to me to see myself as Canadian”, or “it is important to me to see myself as part of my home culture”. For doctors, similar questions referred to their orientations regarding what they expected of their immigrant patients, with questions including such items as “it should be important to them to see themselves as Canadian”, or “it should be important to them to see themselves as part of their home culture”. Answers were on a four-point scale, ranging from “1, completely disagree” to “4, agree completely”.

A clinic visit was then video recorded, which was scheduled at a time when the patient already had a regular visit booked. Clinic visits were recorded with video equipment available in the hospital for residency training, under strict confidentiality agreements.

Interviews were conducted and audio recorded after the video recording, in private offices within the hospital research department, during a convenient time for the doctors and patients, separately. Questions asked to doctors were both specifically related to the patient, and to immigrants on a more general level. These included, for example,“Do you feel confident that this patient agrees your advice is necessary and valuable?”, “Is this true of many of your immigrant patients?”. Questions asked to patients also related specifically to their doctor, and healthcare experiences in general. They included, for example, “did you feel listened to and understood?”, “Were your expectations of the visit met?”

### Analyses

Videos and interviews were transcribed and double-checked by the researcher. Directed content analysis was used, which seeks to ‘validate or extend conceptually a theoretical framework or theory’ [25]. The health consumer/health professional model informed the formulation of our research questions, and guided the creation of our key themes extracted from the interview coding process. We then used the findings to generate hypotheses in support of the health consumer/health professional model. Since directed content analysis has the potential of creating strong bias of the researcher toward the theory guiding the process [25], specific strategical techniques were used for coding the videos and interviews, to ensure analyses were performed systematically with as little bias as possible.

The analytic technique used for videotaped clinic visits was the Verona Coding System [26], a method of specifically examining doctors’ responses to patients’ cues and concerns (for a full schematic diagram, see Appendix A). Each cue (an alluded to or poorly explained concerned is a ‘cue’)/concern (any clearly articulated concern) from the patient elicits a response from the doctor, who either explicitly or non-explicitly addresses the cue/concern. The doctor’s response either provides space for the patient’s cue/concern to be investigated, or reduces space.

Thematic analysis was used for the interviews, to highlight key elements that doctors and patients experienced as most crucial for communication. A six phase process for identifying themes was followed, as outlined by Braun & Clarke [27] (Appendix B). The approach was essentialist/realist (reporting participant’s experiences/meanings), theoretical (driven by the study research questions) and focused on the semantic quality of the data (explicit meanings stated by participants).

AO surveys were analyzed after the interview analysis, so as not to bias interpretation of the videos and interviews. AOs were calculated using the Euclidean Distance Method, which calculates the mean of an individual’s answers on the survey, and then uses the Euclidean Distance formula to plot the distance of each individual’s orientation scores from each of the four extreme orientations (i.e., full assimilation, etc.). This places people with proximity scores toward all of the orientations, to see which they lean toward most. An example calculation can be seen in Appendix C.^2^

The results of the above techniques were compiled and used to interpret whether the themes extracted fit the categories and predictions of the health consumer/health professional model.

## Results

The dominant AOs (for acculturation scores, see Appendix D) and Verona Coding results (number of cues/concerns from the patient/number, number of different responses from the doctor) of each doctor-patient pair are displayed in Table 3. The main interview themes for each doctor-patient pair are displayed in Table 4.

**Table 3:** Verona coding: summary of numbers of patient cues and doctor responses for each doctor-patient pair

Patient	A	B	C	D	E
Orientation	Separation	Integration	Integration	Integration	Integration
Cues	31	53	57	46	33
Concerns	7	1	1	5	7
TOTAL	38	54	58	51	40
Doctor	A	B	C	D	E
Orientation	Separation	Separation	Separation	Marginalization	Marginalization
Providing Space Responses	43	32	49	18	33
Acknowledge content (EPCAc)	23	17	12	12	21
Explore content (EPCEx)	19	15	29	6	10
Acknowledge affect (EPAAc)			3		1
Empathize (EPAEm)	1		1		1
Explore affect (EPAEx)			4		
Reducing Space Responses	32	22	12	43	20
Give information/advice (ERIa)	32	20	11	42	20
Switch subject (ERSw)		2			
Postpone (ERPp)				1	
TOTAL	75	54	61	61	53

**Table 4:** Themes from doctor and patient interviews, and their related content

Doctor A	Doctor B	Doctor C	Doctor D	Doctor E
Challenges in treating immigrants				
Patients often not proactive	Needs more time to talk with/understand	Level of difficulty depends on how long patient was in their previous country	Level of difficulty depends how long they’ve been in Canada	Can be difficult adjusting communication to their level
Not sure they follow her advice	Not always sure they understand what she said and vice versa	Some are compliant but not proactive	Some immigrants have different perspectives on health.	Different approaches to medicine can be hard: many cultures not preventive
Hard to explain different concepts		Difficult to approach topic of background: where to start?		
Hard to advise on psychosocial aspects but they play big role		Unknown daily parts of life that influence health, but neither side thinks to ask about		
Beliefs can conflict				
Expectations				
Unclear what they expect her role to be	Sometimes they don’t follow advice because it’s different in their country	Different expectations of medicine (e.g. cures from pills)	Expects patients to take control of own health.	Sometimes they initially expect same treatment as they get in home country
Understanding				
Education makes a difference to how much they understand	Patients don’t always understand the health system.	Education makes a difference how much the they understand/respond to treatment.	It takes time for patients to understand the healthcare system.	Don’t always understand:
				- role of the doctor
				- health system
Keys to success/ideas for improvement				
More accessible community resources for psychosocial aspects	Adjusts approach depending on integration level	Understand bigger picture of where they come from	Both doctors and patients can adapt and meet in the middle	Establish rapport
	Translators, even if person speaks English	Cultural sensitivity training	Interpreters would be useful	Be open to idea they won’t always take advice
		Questions about culture on health history form		Explain system when they first arrive
Patient A	Patient B	Patient C	Patient D	Patient E
(Philippines)	(China)	(Trinidad)	(Philippines)	(Brazil)
Attitudes/background				
Lived in 3 different countries (Saudi Arabia, Malaysia, Canada)	Eats healthy, tries to get physical activity	Took about 10 yearsto adjust to the culture	Medical advice falls in line with her own beliefs	Lives healthy lifestyle
Follows doctor advice but follows own diet	Believes in needing to adapt to new culture fully and with an open mind	Medical advice falls in line with own beliefs because she reads what’s going on in the country and tries to adapt	Learned to become healthier after pregnancy	Was willing to learn and adapt to Canadian syste
Dismisses difficult experiences with moving: one has to adapt	Hard to adapt at first			People who complain don’t accept that the system is just different
Healthcare experience in home country				
No healthcare in previous countries, have to pay for everything.	Had to pay for everything, much poorer conditions (e.g. no privacy from other patients)	Not as developed	Nothing free, have to pay for everything	Used to going to hospital for everything
		Has pacemaker, wouldn’t have got that in Trinidad		
Healthcare experience in Canada				
Free care	Advice matches own beliefs.	All her needs are taken care of	Her expectations of the treatment are always met	Likes concept of family doctor and having history
		Learned more about health		
Bad experience before present clinic	Problems finding doctor at first	Previous doctor was not attentive or caring	Problems finding doctor at first	Doesn’t like wait times
			Long wait times, would rather pay and not have to wait.	
Experience with present doctor				
Likes doctor: good pep talks very happy	Likes doctor: supportive answers questions	Loves doctor: takes care of her concerns takes time with her	Likes doctor: makes her laugh feel comfortable truly cares	Likes doctor: takes time explains listens

The video codes and interview themes were combined to examine the research questions and generate the following hypotheses:

1. The quality of the doctor-patient relationship and the nature of doctor-patient communication are influenced by the combined AOs of the doctor and patient.
2. a. Patients with an integration attitude, and doctors with any of the four attitudes will report a positive relationship.
3. Patients and doctors who share the same AO will report a consensual relationship.
4. Other AO combinations will report a conflictual/problematic relationship.
5. a. Consensual relationships will positively interrelate with the patients’ and doctors’ personal experiences.
6. Conflictual/problematic relationships negatively interrelate with the patients’ and doctors’ personal experiences.

Further explanation of the derivation of our hypotheses is presented in the discussion section.

## Discussion

This study examined how AOs of doctors and immigrant patients influence their relationship based on the health consumer/health practitioner model (RQ1), and how this interrelates with the experiences of both individuals (RQ2).

While survey scores showed that only one doctor-patient pair matched in their AOs to form a consensual relationship as predicted by the model, all doctors and patients reported a relatively good relationship. According to the health consumer/health practitioner model, however, doctor-patient pairs B, C, D, and E should all have conflictual relationships, which was not the case. It may be that the model is correct in its expectation of AO playing a role in the doctor-patient relationship, but does not accurately predict which combinations produce good working relationships in the specific context of a doctor and immigrant patient interaction. The model predicts that any time a doctor presents a separation or marginalization orientation, the resulting relationship will be conflictual [23]. However, based on the results of our cases, all doctors showed one of these two orientations, without the a resulting conflictual relationship with the patient. This is in contrast to existing literature, which often posits that marginalization and separation generally lead to more negative outcomes [28].

Instead, based on the video and interview information, the separation and marginalization orientations of the doctors manifested as behaviour that was either very culturally sensitive (separation): allowing the immigrants space to maintain their own cultural beliefs, or very culturally neutral (marginalization): understanding that the immigrant may adapt to the host culture or maintain their own, but treating them like any other individual patient regardless of this. Therefore, the separation and marginalization orientations of the doctor did not translate into negative behaviours as expected based on the model, but rather to an acute awareness that a person’s background is complicated, and adaptation or lack of adaption is personal.

Two of the cases involving the combination of a patient with an integration orientation and a doctor with a separation orientation revealed the most patient involvement in terms of discussing cues/concerns. This might have been elicited by the space these doctors give to their patients as a result of the doctors’ cultural sensitivity, which previous literature has found to be comprised of many factors, including effective communication [29]. This could suggest that although AO combinations do not seem to fit the model, it does not mean they are unimportant. The separation and marginalization categories in this particular context seem to translate into slightly different culturally sensitive behaviours, and a positive doctor-patient relationship. Further, the integration attitudes of the patients are a crucial factor in these five relationships, which supports previous literature findings of integration being beneficial for immigrants [11]. This information thus provides some support for the health consumer/health practitioner model, but also a need to reconsider its predictions in this context.

All patients but one showed an integration attitude. One patient showed a separation attitude. However, in her interview her fluency in English was limited and she reported her belief in the importance of adapting to a new culture, so it is possible she did not understand the survey properly. Patients with a separation or marginalization attitude might have exhibited negative behaviours, although this would have to be investigated. An integration attitude likely played a substantial role in the patients being open to doctors’ advice.

This initial evidence for AOs supports literature that has found acculturation to be important in the doctor-patient relationship [30]. We found that relationships between immigrants with integration attitudes, and doctors with marginalization/separation attitudes form reasonably good relationships and positive experiences for both patient and doctor, which led to the hypotheses generated. More detailed studies could investigate the areas of these relationships that continue to be challenging, and how to further improve them through communication and understanding. Differing expectations seems to be an important challenge. Doctors reported that immigrant patients vary in their expectation of the doctor’s role, and how much the doctor should help them to adapt to the new country. The patients in the five observed cases took a substantial amount of responsibility in adapting to their new culture, without expecting their doctor to take care of everything. This is in line with previous findings that patient’s perceptions play a crucial role in the doctor-patient relationship. Immigrant patients with other orientations might show very different behaviour patterns [31].

It should be noted that there are limitations to this study. First, the immigrant patients who participated are probably already better integrated than others, since they visit Canadian doctors and take care of their health. Therefore, none of the patients showed any of the AOs that might lead to a more negative relationship with the doctor. Second, the doctors at St. Mary’s hospital are practicing in a multicultural city, and have already developed an way of with working with immigrants. To further explore the impact of AOs on the doctor-patient relationship, future studies should seek to reach immigrant patients who are not well adapted to the culture, and doctors who are not as familiar with working with immigrant patients. The sample size is small, which also raises some concerns. However, the consistencies seen among these 10 individuals are strong, and suggest that the context may be significant as well: a multicultural hospital, with doctors having a substantial amount of exposure to immgrant patients, revealed doctor-patient pairs reporting good relationships. The findings should still be replicated with larger sample sizes. The gender distribution of the sample could also be considered a limitation, as there were many more females than males. This occurred by chance, since these were the only individuals who had a regular doctor visit scheduled, and agreed to participate. It should be noted, however, that in a usual family practice, roughly 65 % of the visits are with women. Given the small sample size, 80 % being female patients is not very unrepresentative, since women made up 60 % of adult ambulatory care visits in 2012 in the US [32]. For doctors, however, the sample is not representative, as about 50 % of family doctors in the province of Quebec are women [33].

As well, in the doctor-patient pairs, the doctor and patient were always of the same gender, reducing any potential bias. Nonetheless, the study should certainly be replicated with more equal gender distribution.

## Conclusion

Our findings provide some initial support for the predictions made by the health consumer/health practitioner model, namely, that AOs of host culture doctors and immigrant patients play an important role in the quality of the doctor-patient relationship. The key findings revealed from analysis of these interviews included patients showing a willingness to adapt to the new cultures (an integration AO), doctors showing a willingness to accept an immigrant’s culture, and both reporting positive doctor-patient relationships. Since the literature reveals that there are currently many gaps in doctor-immigrant patient communication, which often lead to misunderstandings [7, 13–15], poorer quality of care received [5], and less patient compliance [17, 18], fostering a positive doctor-patient relation has the potential to influence the quality of care, and resulting health of the patient.

This exploratory study opens the door to the notion that AOs between doctors and immigrant patients provide a foundation upon which the doctor-patient relationship is based. Future studies should test the generated hypotheses with stronger analytic methods, in different areas of medicine, in different countries, and possibly eventually in different disciplines.

Many interventions seeking to improve doctor-immigrant patient relationships currently do not follow a standardized method, since not enough knowledge exists currently to create one. Our exploration of AOs found some support of use of the health consumer/health practitioner model to guide the development of effective interventions to improve the quality of doctor-immigrant patient relationships [24]. In this case, interventions based on training and understanding of the influence of AOs could help create a basic level of understanding, and may improve interaction between doctors and immigrant patients.

## Figures and Tables

**Fig. 1:**
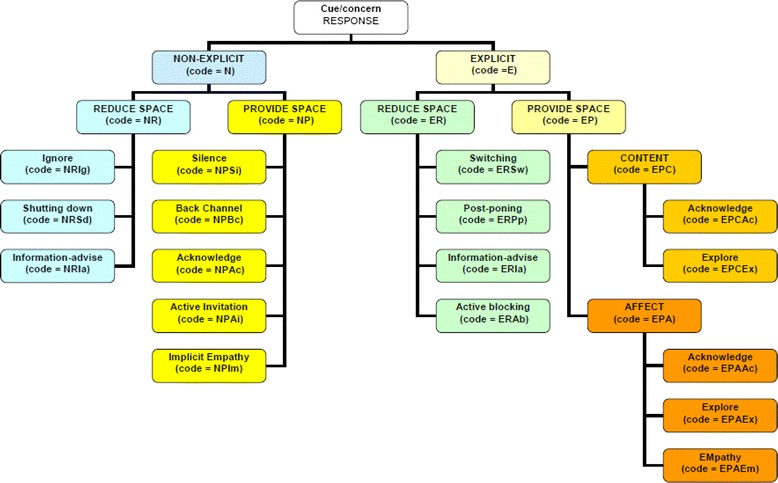
Verona coding system: schematic diagram

## Appendix B

**Table 5:** Six phase process of thematic analysis

Phase	Description of the process
1. Familiarizing yourself with your data:	Transcribing data (if necessary), reading and re-reading the data, noting down initial ideas.
2. Generating initial codes:	Coding interesting features of the data in a systematic fashion across the entire data set, collating data relevant to each code.
3. Searching for themes:	Collating codes in potential themes, gathering all data relevant to each potential theme.
4. Reviewing themes:	Checking if the themes work in relation to the coded extracts (Level 1) and the entire data set (Level 2), generating a thematic ‘map’ of the analysis.
5. Defining and naming themes:	Ongoing analysis to refine the specifics of each theme, and the overall story the analysis tells, generating clear definitions and names for each theme.
6. Producing the report	The final opportunity for analysis. Selection of vivid, compelling extract examples, final analysis of selected extracts, relating back of the analysis to the research question and literature, producing a scholarly report of the analysis.

## Appendix C – Euclidean distance calculation example: Patient A

**Scores:**

Completely disagree = 1

Slightly disagree = 2

Slightly agree = 3

Agree completely = 4

**Mean of answers to questions about home culture:** 2.86

**Mean of answers to questions about host culture (Canada):** 2.43

**Calculate Distance Score from each orientation with Euclidean Distance Formula:**

$$ \mathrm{D}\left(\mathrm{x},\ \mathrm{y}\right) = \surd\ {\left({\mathrm{x}}_1\hbox{--}\ {\mathrm{y}}_1\right)}^2 + {\left({\mathrm{x}}_2\hbox{--}\ {\mathrm{y}}_2\right)}^2 $$

X_1_ = mean score on questions about home culture

X _2_ = mean score on questions about host culture

Y_1_ and Y_2_ = Most extreme scores for each orientation (Marginalization = 1, 1; Separation = 4, 1; Assimilation = 1, 4; Integration = 4, 4).

**Distance Scores for Patient A:**

From full Marginalization: √ (2.86 − 1)^2^ + (2.43 − 1)^2^ = √ 5.5 = 2.35

From full Separation: √ (2.86 − 4)^2^ + (2.43 − 1)^2^ = √ 3.34 = 1.83

From full Assimilation: √ (2.86 − 1)^2^ + (2.43 − 4)^2^ = √ 5.92 = 2.43

From full Integration: √ (2.86 − 4)^2^ + (2.43 − 4)^2^ = √ 3.76 = 1.93

***Distance scores can range from 0 to 4.24. Proximity scores to each orientation are then calculated:**

Marginalization: 4.24−2.35 = 1.89

Separation: 4.24−1.83 = 2.41

Assimilation: 4.24−2.43 = 1.81

Integration: 4.24−1.93 = 2.31

***These scores are used to plot the person in two dimensional space, and observe visually toward which orientation they lean the most***

## Appendix D

**Table 6:** Table of calculated acculturation orientation scores for doctors and patiens. Acculturation orientations (AO) of doctors and patients: highest scores indicate which orientation individuals lean towards the most, but are not completely exclusive of other orientations

Proximity Score	Doctor A	Doctor B	Doctor C	Doctor D	Doctor E
Marginalization	1.61	1.61	1.64	2.41	2.57
Separation	2.77	2.26	3.64	2.30	2.45
Assimilation	1.38	1.20	0.61	1.89	1.67
Integration	2.39	1.74	1.64	1.81	1.59
Proximity Score	Patient A	Patient B	Patient C	Patient D	Patient E
Marginalization	1.89	1.81	1.70	1.41	1.70
Separation	2.41	1.89	2.38	2	2.38
Assimilation	1.81	2.29	1.78	2	1.78
Integration	2.31	2.42	2.50	2.82	2.50

As can be seen above, Doctor and Patient A are the only pair who have a combination of acculturation orientations that match to provide consensual relationships as predicted by the health practitioner/health consumer model. The other four doctor/patient pairs show orientation combinations that lead to conflictual/problematic relationships according to the health practitioner/health consumer model

## Footnotes

1 These orientations can refer to the immigrant’s attitudes about him or herself, *or*, a host culture member’s orientation in terms of what he or she *expects* of an individual immigrant or immigrant population. Here, acculturation orientation is based on this model, conceptualized in Table 1.

2 The entire quantitative aspect of the study included 171 participants, thus providing an adequate sample size for such analyses. The 10 quantitative acculturation orientation calculations reported in this paper are not to be taken as a full scale quantitative analyses, but rather as a first indication of the quantitative method used, which will be presented in later stages.
